# Demographic and Clinical Characteristics of Patients Vaccinated Against Herpes Zoster in a Primary Care Setting in Spain: A Retrospective Study

**DOI:** 10.3390/vaccines13101069

**Published:** 2025-10-20

**Authors:** José María Blanc-Rodriguez-Arias, Isabel Jimeno-Sanz, Valentín Hernández-Barrera, Alejandro Álvaro-Meca, Manuel Torres-Ramos, Ángel Gil-de-Miguel

**Affiliations:** 1 Department of Medical Specialties and Public Health, Area of Preventive Medicine and Public Health, Rey Juan Carlos University, 28916 Madrid, Spain; 2 Primary Care Health Centre Isla de Oza, 28035 Madrid, Spain; 3 Family and Community Medicine, Jimenez-Diaz Foundation, 28040 Madrid, Spain

**Keywords:** herpes zoster, vaccination, epidemiology, comorbidities, adult immunisation

## Abstract

Background: Herpes zoster (HZ), commonly known as shingles, is a reactivation of the varicella-zoster virus that predominantly affects older adults and immunocompromised individuals. Vaccination with the recombinant zoster vaccine (RZV) has proven effective in preventing HZ and its complications in real-world scenarios, yet coverage remains suboptimal in primary care settings. This study aims to analyse the demographic and clinical characteristics of HZ-vaccinated patients in a primary care centre in Madrid, Spain, in 2023 and to assess adherence to the vaccination schedule. Methods: A retrospective descriptive analysis was conducted, including 1146 patients from a single primary care centre who received at least one dose of RZV during the study period. Data were extracted from medical records. Patient characteristics, including age, sex, and the presence of comorbidities (e.g., diabetes, chronic obstructive pulmonary disease, hypertension, asthma), were examined. Vaccine coverage was calculated for the new age cohorts introduced in the year 2023. Comorbidity prevalence was also analysed by age and sex to determine patterns in vaccination uptake. Results: The analysis revealed that 66% of vaccinated individuals presented one or more of the analysed comorbidities. Adherence to the two-dose schedule was 91.97%, and vaccine coverages were 46.56% in those born between 1943–1948 and 39.32% in the 1958 cohort. The findings underscore the need for targeted strategies to raise disease awareness and improve HZ vaccination rates and adherence, particularly among elderly individuals and those with comorbid conditions. Conclusions: This study specifies the clinical characteristics of HZ-vaccinated patients in a primary care setting and highlights the importance of enhanced vaccination awareness and adherence strategies to reduce the burden of HZ among at-risk populations.

## 1. Introduction

Herpes zoster (HZ), commonly known as shingles, is a disease caused by the reactivation of the latent varicella-zoster virus (VZV) in individuals who have been previously exposed to this pathogen [1]. VZV primary infection usually happens during childhood, causing chickenpox, and is recognised by a vesicular eruption that extends all over the body in the form of small vesicles [1]. After primary infection, VZV remains latent in sensory neurons in the dorsal root and trigeminal ganglia, and this infection subsequently induces cellular immunity, which impedes reactivation of the virus [2].

HZ is characterised by the appearance of a painful rash and the subsequent formation of vesicles, which normally dry approximately 10–14 days after their appearance. An episode of HZ usually resolves after 4–6 weeks, but it is associated with a high probability of developing complications [1,3]. The most common of these complications is postherpetic neuralgia (PHN), which is defined as pain persisting for more than 3 months after the appearance of the lesions [4]. Other complications range from ophthalmic to neurological or cardiovascular effects [5].

The decline in cellular immunity against latent VZV appears to be responsible for the reactivation of the virus and the subsequent appearance of HZ [6]. This explains the increased risk that older adults and immunocompromised patients have of developing HZ, since these populations usually suffer an impairment of their immune response [7,8,9].

The vulnerability of older adults to infections is partly caused by immunosenescence, which is defined as an age-related decline in immune function [10]. This waning of the immune function also explains the fact that vaccination usually has a lower efficacy in older populations [10,11]. Immunosenescence affects mostly the adaptive immune system in both the cellular and humoral response. The cellular response is weakened through a decline in the overall number of naïve T cells and through an accumulation of functional defects in the available pool of naïve T cells [10,11]. The number of naïve B cells is also diminished in elderly individuals, which might be a cause of the deficient response to vaccination in this population [10,11].

Patients with comorbidities and chronic conditions, such as diabetes, rheumatoid arthritis, cardiovascular diseases, asthma, chronic obstructive pulmonary disease (COPD) or depression, have been shown to be at increased risk of developing HZ, and this additional condition can have a severe impact on their quality of life [12,13]. As older adults are also more prone to the development of comorbidities, this combination of factors makes them particularly vulnerable to the development of HZ and its complications [9].

Several observational studies have reported a higher risk of myocardial infarction, stroke and Guillain-Barré syndrome after a HZ episode [14,15,16,17]. The existence of underlying comorbidities and chronic conditions has also been shown to increase the risk of developing HZ. In the case of COPD, the risk of HZ has been shown to increase by 45% in patients with COPD and by no less than 61% among patients with COPD receiving treatment with inhaled corticosteroids (ICS) with respect to the non-COPD population [18]. Moreover, patients with COPD who suffer an episode of HZ have a greater hospitalisation risk, a greater average number of outpatient visits and overall greater utilisation of health care resources [18]. Surprisingly, we found that the vaccination rate of patients with COPD was significantly higher in individuals in the 50–64 years cohort than in patients of more advanced age. This may indicate greater awareness of HZ and its complications in this age group than in older patients, especially given that these patients are not generally included in the systematic vaccination schedule financed by the Spanish Ministry of Health. Patients with asthma are also at increased risk (36% increase in patients > 50) of developing HZ and its complications [19].

Patients with diabetes are also at a greater risk of HZ infection, with the adjusted risk rising by 20% among people with diabetes in comparison with the nondiabetic population [20]. Interestingly, these studies also revealed that the relationships between HZ and underlying comorbidities such as COPD, asthma or diabetes are bidirectional. Not only are these patients at a greater risk of developing HZ, but an episode of HZ may also contribute to a worsening of their underlying conditions, which usually results in a greater utilisation of health care resources and a severe impact on their quality of life [18,20].

HZ has been widely described as a disease that takes a heavy toll on both functionality and quality of life and interferes with daily activities, sleep, and general mood, among other factors [12]. Moreover, the global incidence of HZ is increasing as life expectancy increases worldwide, and the number of older adults increases annually [21].

A systematic literature review estimated an annual incidence between 5.23 and 10.9 cases per 1000 person-years worldwide. Cumulative incidence was estimated to be between 2.9 and 19.5 cases per 1000 people worldwide [21]. This study found that globally, cumulative incidence and incidence rates are higher in females.

Between 2014 and 2018, the incidence of HZ in Spain was estimated to be 351.6/100,000 inhabitants, with 625.5/100,000 in the population > 50 years old [22]. The incidence rate increases with age, particularly in the 50–54 age group (a 41% increase over the 45–49 age group). With respect to hospitalisation rates (HR) due to HZ, there is also a marked increase with age, especially from the 60–64 years onwards, with a 50% increase compared with younger cohorts [22]. In the immunocompromised population, the incidence rate of HZ complications is estimated to be approximately 5 times higher than that in the healthy population, which results in more frequent and severe HZ episodes and therefore in greater utilisation of health care resources [7]. Previous studies have analysed the burden of hospitalisation in Spain [23,24]. During the COVID-19 pandemic (2020–2021), the hospitalisation rate (HR) was found to be 14.4 cases per 100,000 inhabitants, and the mortality rate (MR) was 1.3 cases per 100,000 inhabitants. Notably, 92.3% of registered hospitalisations were in patients >50 years old, and costs due to HZ disease hospitalisation during the studied period were estimated at 100,433,904 € [24].

The standard treatment for the acute phase of an HZ infection is antiviral therapy, which works by inhibiting viral replication [4,25]. In addition, different analgesic medications should be administered depending on the intensity of pain [26,27]. The recommended treatments for PHN-related pain include opioids, α_2_δ-ligand antiepileptics (such as gabapentin or pregabalin) and tricyclic antidepressants [26]. With respect to PHN prevention, antiviral therapy appears to have some effectiveness if the treatment is initiated within 72 h of the appearance of the rash [26]. However, the efficacy of antivirals in the prevention of PHN is limited Vaccination currently seems to be the most effective strategy for preventing HZ and PHN [26,28].

Currently, there is only one HZ vaccine marketed in Spain, an adjuvant recombinant zoster vaccine (RZV, Shingrix, GlaxoSmithKline Biologics). This is a subunit vaccine with a two-dose administration schedule [29]. In March 2021, the Interterritorial Council of the National Health System (Consejo Interterritorial del Sistema Nacional de Salud, CISNS) recommended systematic HZ vaccination with RZV for all adults turning 65 and 80 years, with an instruction to progressively vaccinate those aged between 66 and 80 years [30]. Following national guidance, the region Madrid prioritised routine HZ vaccination with recombinant zoster vaccine (RZV) for specific birth cohorts. In 2023 the publicly financed cohorts were those born between 1943–1948 and those born in 1958. In addition, vaccination is publicly financed for defined risk groups (transplant recipients, malignant hematologic disease, solid tumours under chemotherapy, HIV infection, patients on JAK inhibitors, patients under other immunomodulatory/immunosuppressive treatments, people who have already suffered 2 or more previous episodes of HZ), in line with national recommendations [30]. Outside these prioritised cohorts and risk groups, vaccination may be recommended but not publicly financed, depending on regional implementation [31].

RZV has shown positive efficacy and safety data in two phase III clinical trials with immunocompetent adults aged >50 years and >70 years [32,33]. In these clinical trials, the efficacy of two RZV doses was above 89% for all age groups, and serious adverse events occurred with similar frequencies in both the placebo group and the RZV group [32,33]. In addition, several studies have been published that demonstrate the effectiveness of RZV in a real-world setting. In these real-world studies, RZV has been shown to be effective against HZ oscillating between 70.1% and 86.7% and between 51.4% and 76% against PHN. These studies included heterogeneous populations, such as older adults, immunocompromised patients, patients with comorbidities and patients with autoimmune diseases, and the administration did not necessarily follow the 0–2 month schedule [34,35,36,37,38,39]. RZV vaccination has also been shown to have a positive effect on patients’ quality of life. This is mainly due to the high efficacy it has demonstrated, which prevents a significant number of patients from experiencing the burden of HZ and complications such as PHN, which take a heavy toll on daily life, especially in older adults and patients with underlying conditions [40]. Moreover, it has been demonstrated that in breakthrough cases of HZ in people who have received the vaccine, patients are less likely to experience intense pain, and this pain tends to be shorter in duration [40]. In addition, they also reported higher quality of life scores and less interference in their daily life activities [40]. Vaccination against HZ has also recently been associated with a decrease in the risk of developing dementia suggesting a causal relationship between HZ infection and neuroinflammation, which may lead to a neurodegenerative state [41,42].

A recent study assessing the public health impact of vaccinating adults > 50 years with RZV in Spain revealed that RZV could prevent up to 1,533,353 HZ cases and 261,610 PHN episodes [43]. Moreover, it is estimated that systematic HZ vaccination in adults > 50 years could prevent 71,156 hospitalisations and 3,500,492 primary care visits, which would drastically reduce health care resource usage [43].

The objective of this study is to describe the profile of adults vaccinated against HZ in a primary care centre in Madrid during 2023 and assess adherence to the two-dose RZV schedule. The studied profile factors were selected based on prior associations with HZ risk and vaccine targeting in guidance: age, sex, and comorbidities (diabetes, COPD, asthma, hypertension, rheumatoid arthritis, depression, HIV, and prior HZ episode).

## 2. Materials and Methods

We conducted a retrospective descriptive analysis of patients vaccinated against herpes zoster (HZ) in a single public primary care centre in Madrid from 1 January–31 December 2023. The centre’s assigned population comprising 20,828 patients, who receive medical assistance beyond vaccination at this site. The total population numbers that belong to this health centre, as well as the age distribution, were provided by the centre’s General Direction. The centre’s population distribution by age group can be found on Table 1. In our analysis, we included all adults in the assigned population who received ≥1 RZV dose in 2023 at the centre (vaccinated cohort). The following variables were assessed: age, sex, and the presence of specific comorbidities known to increase HZ risk based on guidance and literature: diabetes, asthma, COPD, HIV, rheumatoid arthritis, a previous episode of HZ, hypertension, and depression. Diagnoses for these conditions correspond to medical diagnoses from health care providers.

Data were collected from computerised health records from patients obtained at the primary care centre. Data were extracted taking into account demographic and clinical information relevant to HZ. For coverage calculations (limited to programmed cohorts in 2023), we used counts of eligible persons (born 1943–1948 and 1958) as denominators. Individuals whose first dose occurred after 30 October 2023 (i.e., within 8 weeks of year-end) were excluded from coverage and completion-rate denominators. Categorical variables were summarised as *N* (%). To identify differences in the clinical profile of vaccinated patients, we compared the prevalence of each comorbidity between men and women, and across age groups, using the Chi-squared test or Fisher’s exact test as appropriate. Vaccine coverage rates were calculated for the eligible cohorts, with 95% confidence intervals estimated using the binomial method. Statistical significance was set at *p* < 0.05. All analyses were performed using SPSS 29.0 (IBM).

The health centre in which the study was conducted has a total of 15 consultation rooms and is staffed with 12 general practitioners (GPs), 2.5 paediatricians, 10 nurses and 7 administrative staff.

## 3. Results

A total of 1146 patients were vaccinated against HZ in this health centre during the 12 months of the study period. A total of 870 patients received the full two-dose schedule, and 276 received only the first dose by the end of the study, of which 76 had received it more than 8 weeks prior to the end of the study. This translates to an adherence rate of 91.97% to the vaccination schedule during the analysed period. The distribution by sex of the 1146 patients was 451 men (43.16%) and 695 women (56.84%).

### 3.1. Vaccinated Patients by Age Group

With the data of the total population of this health centre distributed by age group, we determined the number of vaccinated patients in each of the age groups eligible for HZ vaccination in this health centre in 2023, as shown in Table 2.

### 3.2. Vaccine Coverage

Vaccine coverage has been calculated for the new cohorts included in the year 2023 (in Madrid, those individuals born in 1943–1948 and 1958). The results are shown in Table 3.

### 3.3. Comorbidities of Vaccinated Individuals by Age Group

**Figure 1:**
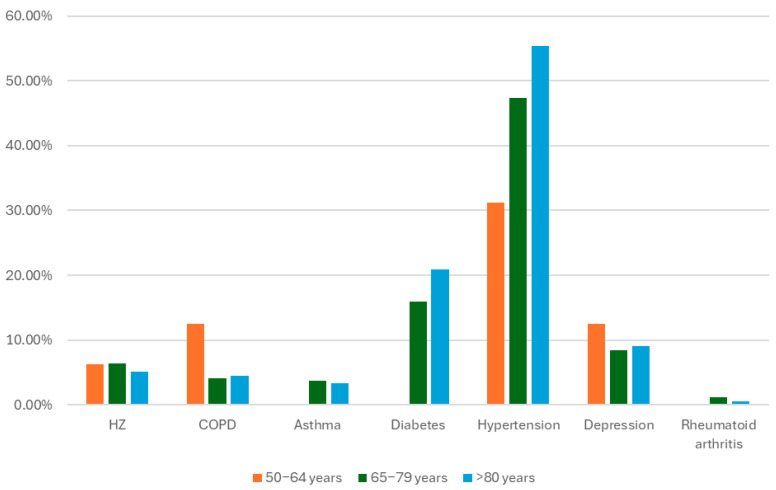
Prevalence of comorbidities in vaccinated patients by age group.

The percentage of vaccinated individuals with one or more comorbidities was high (66%) in the studied population. In some cases, the occurrence of comorbidities increases with age. For example, the percentage of patients with type II diabetes increased from 15.95% in the 65–79 years group to 20.90% in the ≥80 years group. Another example is hypertension, which affects 47.32% of individuals in the 65–79 years group and 55.37% in the ≥80 years group. However, the prevalence of previous episodes of HZ was very similar among all age groups, as was the prevalence of depression. However, the populations of the 50–64-year-old group and the ≥80-year-old group were much smaller than those of the 65–79-year-old group. The prevalence of COPD was significantly greater in the 50–64-year group (12.50%) than in the 65–79-year group (4.09%) and the ≥80-year group (4.52%). Figure 1 illustrates the prevalence of the different comorbidities distributed by age groups.

### 3.4. Comorbidities of Vaccinated Individuals by Sex

**Figure 2:**
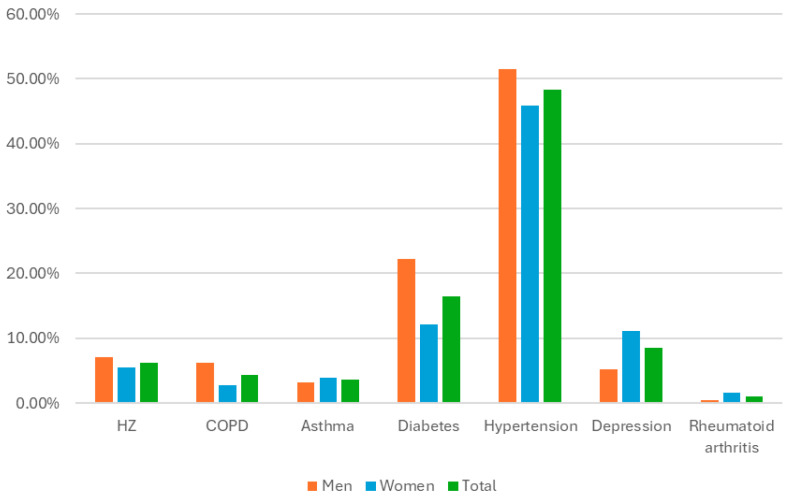
Prevalence of comorbidities by sex.

An analysis of the distribution of the different comorbidities in relation to the sex of the individuals revealed that, even though female sex is listed as an independent risk factor for HZ [7,44], we found that the HZ rates were very similar between the sexes and were even slightly higher in men (7.07% vs. 5.52% in women). The prevalence of COPD was significantly greater in men (6.26%) than in women (2.76%), as was the prevalence of diabetes (22.22% in men vs. 12.12% in women). The prevalence of depression, however, was greater in women (11.04%) than in men (5.25%). These findings are summarised in Table 4 and Figure 2.

## 4. Discussion

In this descriptive study, we analysed the average clinical profile of patients vaccinated against herpes zoster in a primary care setting in 2023 in Spain and their adherence pattern to the two-dose schedule. During the time of the study, we gathered data from 1146 patients who had received the vaccine regarding their age, sex and presence of several comorbidities associated with an increased risk of developing HZ (asthma, COPD, diabetes, hypertension, rheumatoid arthritis, depression, and HIV). We found that nearly two-thirds (66%) of the patients vaccinated against HZ in 2023 had at least one of these comorbidities. The coverage rates for the full two-dose schedule in the cohorts included in 2023 were acceptable with 39.32% in the 65 years cohort and 46.56% in the 75–80 age group. The rate of adherence to the two-dose schedule by the end of the study period was 91.97%. This is an interesting finding, especially since completion of the full schedule is crucial to achieve long-term protection against HZ and PHN. The completion of the full vaccination schedule is of particular importance, since while vaccine effectiveness is high after the first dose is administered, RWE studies have demonstrated that it sharply decreases 12 months after administration if the second dose is not administered [39]. Coverage data are far from optimal and suggest the need for better awareness campaigns aimed at those who are included in the eligible population every year (i.e., those turning 65 and 75 years old). These coverage rates are below what can be observed in relation to other adult vaccine-preventable diseases (VPDs) in the region of Madrid. For instance, vaccination coverage against influenza in individuals aged ≥65 during the seasons 2022–2023 and 2023–2024 was at around 70% according to a report published by regional health authorities [45]. Regarding pneumococcal disease, coverage in the cohort included in the year 2022 was 51.2% [45].

A 2025 report states that 88.5% of individuals vaccinated against HZ in Madrid have been so in a primary care setting [31]. This finding highlights the key role that primary care plays on vaccination and protecting the population from the burden of infectious diseases. Therefore, medical education initiatives targeted towards primary care health care providers (HCPs) about the risk of adult VPDs in general and HZ in particular, as well as the value of adult immunisation as a part of healthy ageing.

Another strategy that has proven useful is the implementation of disease awareness campaigns in primary care centres that may reach the general public so individuals in the recommended age cohorts may proactively seek to be vaccinated against HZ and other adult VPDs. These strategies would undoubtedly contribute to an increase in coverage and therefore to protect a larger number of patients from the burden associated with HZ and its complications.

In the light of the findings of this study regarding the clinical profile of patients vaccinated in primary care in Spain, it would be interesting to analyse the feasibility of including people with certain chronic conditions (such as COPD, diabetes, depression or people with 1 previous episode of HZ) in the publicly financed groups that may receive this vaccine, as some scientific societies have suggested, given the increased risk these patients have of suffering from an episode of HZ.

This study has certain limitations that should be acknowledged. The retrospective design relies on the accuracy of medical records, which could lead to minor data omissions or inaccuracies. Additionally, the study is limited by its local scope in a single primary care centre, which may not reflect the demographic and clinical characteristics of patients in other regions. The relatively small sample size further limits the generalisability of the findings. Despite these limitations, the study provides valuable insights into vaccination adherence and coverage in a real-world primary care setting, offering a strong foundation for further research and public health interventions. Future studies should aim to include both vaccinated and unvaccinated populations to better understand the drivers of vaccination decisions in primary care settings.

## 5. Conclusions

The results from this study show that even though patients vaccinated against HZ in a primary health care setting fit the clinical profile established by public health recommendations, vaccine coverage is still not optimal. The achievement of greater vaccination coverage is essential to avoid the burden of HZ and PHN on older adults, which can have a severe impact on their quality of life as well as on their preexisting comorbidities. Compliance with the two-dose schedule is also key to optimise protection in vaccinated individuals. There is a need for implementation of public health interventions designed to raise awareness about HZ and its complications among both health care professionals and general public. While these results provide useful insights into adherence and coverage in a local setting, further multicentre studies with larger populations are needed to confirm these findings at a regional or national level.

## Figures and Tables

**Table 1:** Health centre population by age group.

Age	<2	2–3	4–6	7–13	14–49	50–64	65–79	>80	Total
Number	268	279	376	1046	9187	4229	3948	1495	20,828

**Table 2:** Vaccinated population by age group during the study period.

Age Group	Vaccinated Population
50–64 years	16
65–79 years	953
80 years or more	177
Total	1146

**Table 3:** Vaccine coverage in cohorts included in 2023.

Age	Total Eligible Population	Vaccinated Population	Coverage
65	206	87	39.32%
75–80	1351	629	46.56%

**Table 4:** Prevalence of comorbidities by sex.

Comorbidity	Men	Women	Total
HZ	7.07%	5.52%	6.19%
COPD	6.26%	2.76%	4.27%
Asthma	3.23%	3.83%	3.57%
Diabetes	22.22%	12.12%	16.48%
Hypertension	51.52%	45.86%	48.30%
Depression	5.25%	11.04%	8.54%
Rheumatoid arthritis	0.40%	1.53%	1.05%

## Data Availability

The datasets analysed during the current study are available from the corresponding author, Jose María Blanc Rodríguez-Arias, on reasonable request.

## References

[1] Mueller N.H., Gilden D.H., Cohrs R.J., Mahalingam R., Nagel M.A. (2008). Varicella zoster virus infection: Clinical features, molecular pathogenesis of disease, and latency. Neurol. Clin..

[2] Kennedy P.G. (2002). Varicella-zoster virus latency in human ganglia. Rev. Med. Virol..

[3] Oxman M.N. (2009). Herpes zoster pathogenesis and cell-mediated immunity and immunosenescence. J. Am. Osteopath Assoc..

[4] Dworkin R.H., Johnson R.W., Breuer J., Gnann J.W., Levin M.J., Backonja M., Betts R.F., Gershon A.A., Haanpää M.L., McKendrick M.W. (2007). Recommendations for the management of herpes zoster. Clin. Infect. Dis..

[5] Kennedy P.G.E., Gershon A.A. (2018). Clinical Features of Varicella-Zoster Virus Infection. Viruses.

[6] Arvin A.M. (2008). Humoral and cellular immunity to varicella-zoster virus: An overview. J. Infect. Dis..

[7] Munoz-Quiles C., Lopez-Lacort M., Diez-Domingo J., Orrico-Sanchez A. (2020). Herpes zoster risk and burden of disease in immunocompromised populations: A population-based study using health system integrated databases, 2009–2014. BMC Infect. Dis..

[8] Johnson R.W., Wasner G., Saddier P., Baron R. (2008). Herpes zoster and postherpetic neuralgia: Optimizing management in the elderly patient. Drugs Aging..

[9] Marra F., Parhar K., Huang B., Vadlamudi N. (2020). Risk Factors for Herpes Zoster Infection: A Meta-Analysis. Open Forum Infect. Dis..

[10] Weiskopf D., Weinberger B., Grubeck-Loebenstein B. (2009). The aging of the immune system. Transpl. Int..

[11] Crooke S.N., Ovsyannikova I.G., Poland G.A., Kennedy R.B. (2019). Immunosenescence and human vaccine immune responses. Immun. Ageing..

[12] Drolet M., Brisson M., Schmader K.E., Levin M.J., Johnson R., Oxman M.N., Patrick D., Blanchette C., Mansi J.A. (2010). The impact of herpes zoster and postherpetic neuralgia on health-related quality of life: A prospective study. CMAJ.

[13] Lang P.O., Aspinall R. (2021). Vaccination for quality of life: Herpes-zoster vaccines. Aging Clin. Exp. Res..

[14] Marra F., Ruckenstein J., Richardson K. (2017). A meta-analysis of stroke risk following herpes zoster infection. BMC Infect. Dis..

[15] Parameswaran G.I., Drye A.F., Wattengel B.A., Carter M.T., Doyle K.M., Mergenhagen K.A. (2023). Increased Myocardial Infarction Risk Following Herpes Zoster Infection. Open Forum Infect. Dis..

[16] Parameswaran G.I., A Wattengel B., Chua H.C., Swiderek J., Fuchs T., Carter M.T., Goode L., Doyle K., A Mergenhagen K. (2023). Increased Stroke Risk Following Herpes Zoster Infection and Protection With Zoster Vaccine. Clin. Infect. Dis..

[17] Anderson T.C., Leung J.W., Harpaz R., Dooling K.L. (2021). Risk of Guillain-Barre syndrome following herpes zoster, United States, 2010–2018. Hum. Vaccin Immunother..

[18] Munoz-Quiles C., Lopez-Lacort M., Diez-Domingo J. (2018). Risk and impact of herpes zoster among COPD patients: A population-based study, 2009–2014. BMC Infect. Dis..

[19] Mortimer K.J., Cruz A.A., Sepulveda-Pachon I.T., Jorga A., Vroling H., Williams C. (2024). Global herpes zoster burden in adults with asthma: A systematic review and meta-analysis. Eur. Respir. J..

[20] Munoz-Quiles C., Lopez-Lacort M., Ampudia-Blasco F.J., Diez-Domingo J. (2017). Risk and impact of herpes zoster on patients with diabetes: A population-based study, 2009–2014. Hum. Vaccin Immunother..

[21] van Oorschot D., Vroling H., Bunge E., Diaz-Decaro J., Curran D., Yawn B. (2021). A systematic literature review of herpes zoster incidence worldwide. Hum. Vaccin Immunother..

[22] Masa-Calles J., Lopez-Perea N., Vila Cordero B., Carmona R. (2021). [Surveillance and epidemiology of Herpes Zoster in Spain]. Rev. Esp. Salud Publica.

[23] Corcuera-Munguia M., Gil-Prieto R., Garcia-Carretero R., Gil-de-Miguel A. (2023). Hospitalization Burden Related to Herpes Zoster Infection in Spain (2016–2019). Infect. Dis. Ther..

[24] Irigoyen-Mansilla V.M., Gil-Prieto R., Gea-Izquierdo E., Barrio-Fernandez J.L., Hernandez-Barrera V., Gil-de-Miguel A. (2023). Hospitalization burden related to herpes zoster infection during the COVID-19 pandemic in Spain (2020–2021). Hum. Vaccin Immunother..

[25] Gnann J.W., Arvin A., Campadelli-Fiume G., Mocarski E., Moore P.S., Roizman B., Whitley R., Yamanishi K. (2007). Antiviral therapy of varicella-zoster virus infections. Human Herpesviruses: Biology, Therapy, and Immunoprophylaxis.

[26] Saguil A., Kane S., Mercado M., Lauters R. (2017). Herpes Zoster and Postherpetic Neuralgia: Prevention and Management. Am. Fam. Physician..

[27] Werner R.N., Nikkels A., Marinović B., Schäfer M., Czarnecka-Operacz M., Agius A., Bata-Csörgő Z., Breuer J., Girolomoni G., Gross G. (2017). European consensus-based (S2k) Guideline on the Management of Herpes Zoster-guided by the European Dermatology Forum (EDF) in cooperation with the European Academy of Dermatology and Venereology (EADV), Part 1: Diagnosis. J. Eur. Acad. Dermatol. Venereol..

[28] Bruxelle J., Pinchinat S. (2012). Effectiveness of antiviral treatment on acute phase of herpes zoster and development of post herpetic neuralgia: Review of international publications. Med. Mal. Infect..

[29] Dooling K.L., Guo A., Patel M., Lee G.M., Moore K., Belongia E.A., Harpaz R. (2018). Recommendations of the Advisory Committee on Immunization Practices for Use of Herpes Zoster Vaccines. Morb. Mortal. Wkly. Rep..

[30] Grupo de Trabajo de Vacunación Frente a Herpes Zóster de la Ponencia de Programa y Registro de Vacunaciones (2021). Comisión de Salud Pública del Consejo Interterritorial del Sistema Nacional de Salud. Ministerio de Sanidad.Recomendaciones de Vacunación Frente a Herpes Zóster. https://www.mscbs.gob.es/profesionales/saludPublica/prevPromocion/vacunaciones/programasDeVacunacion/docs/HerpesZoster_RecomendacionesVacunacion.pdf.

[31] (2025). Madrid. DGdSPCdSCd. Informe de Seguimiento de la Vacunación Frente a Herpes Zóster en la Comunidad de Madrid. https://www.comunidad.madrid/sites/default/files/doc/sanidad/prev/informe_seguimeinto_vacunacion_hz.pdf.

[32] Cunningham A.L., Lal H., Kovac M., Chlibek R., Hwang S.-J., Díez-Domingo J., Godeaux O. (2016). Efficacy of the Herpes Zoster Subunit Vaccine in Adults 70 Years of Age or Older. N. Engl. J. Med..

[33] Lal H., Cunningham A.L., Godeaux O., Chlibek R., Diez-Domingo J., Hwang S.-J., Levin M.J. (2015). Efficacy of an adjuvanted herpes zoster subunit vaccine in older adults. N. Engl. J. Med..

[34] Izurieta H.S., Wu X., Forshee R., Lu Y., Sung H.-M., Agger P.E., Chillarige Y., Link-Gelles R., Lufkin B., Wernecke M. (2021). Recombinant Zoster Vaccine (Shingrix): Real-World Effectiveness in the First 2 Years Post-Licensure. Clin. Infect. Dis..

[35] Sun X., Wei Z., Lin H., Jit M., Li Z., Fu C. (2021). Incidence and disease burden of herpes zoster in the population aged >/=50 years in China: Data from an integrated health care network. J. Infect..

[36] Sun Y., Jackson K., Dalmon C.A., Shapiro B.L., Nie S., Wong C., Arnold B.F., Porco T.C., Acharya N.R. (2021). Effectiveness of the recombinant zoster vaccine among Kaiser Permanente Hawaii enrollees aged 50 and older: A retrospective cohort study. Vaccine.

[37] Sun Y., Kim E., Kong C.L., Arnold B.F., Porco T.C., Acharya N.R. (2021). Effectiveness of the Recombinant Zoster Vaccine in Adults Aged 50 and Older in the United States: A Claims-Based Cohort Study. Clin. Infect. Dis..

[38] Florea A., Sy L., Qian L., Ackerson B., Luo Y., Wu J., Cheng Y., Ku J., Vega Daily L., Takhar H. (2024). Real-world effectiveness of recombinant zoster vaccine in self-identified Chinese individuals aged >/=50 years in the United States. Hum. Vaccin Immunother..

[39] Zerbo O., Bartlett J., Fireman B., Lewis N., Goddard K., Dooling K., Duffy J., Glanz J., Naleway A., Donahue J.G. (2024). Effectiveness of Recombinant Zoster Vaccine Against Herpes Zoster in a Real-World Setting. Ann. Intern. Med..

[40] Curran D., Oostvogels L., Heineman T., Matthews S., McElhaney J., McNeil S., Diez-Domingo J., Lal H., Andrews C., Athan E. (2019). Quality of Life Impact of an Adjuvanted Recombinant Zoster Vaccine in Adults Aged 50 Years and Older. J. Gerontol. A Biol. Sci. Med. Sci..

[41] Shah S., Dahal K., Thapa S., Subedi P., Paudel B.S., Chand S., Salem A., Lammle M., Sah R., Krsak M. (2024). Herpes zoster vaccination and the risk of dementia: A systematic review and meta-analysis. Brain Behav..

[42] Taquet M., Dercon Q., Todd J.A., Harrison P.J. (2024). The recombinant shingles vaccine is associated with lower risk of dementia. Nat. Med..

[43] Garcia A., Vallejo-Aparicio L.A., Cambronero Martinez R. (2024). The public health impact of recombinant herpes zoster vaccination in adults over 50 years in Spain. Hum. Vaccin Immunother..

[44] Opstelten W., Van Essen G.A., Schellevis F., Verheij T.J., Moons K.G. (2006). Gender as an independent risk factor for herpes zoster: A population-based prospective study. Ann. Epidemiol..

[45] Pública (2024). CdMDGdS. Informe de Coberturas de Vacunación. https://www.comunidad.madrid/sites/default/files/doc/sanidad/prev/informe_coberturas_de_vacunacion_cm_2019_2023.pdf.

